# Inhibition of mitochondrial fission activates glycogen synthesis to support cell survival in colon cancer

**DOI:** 10.1038/s41419-023-06202-3

**Published:** 2023-10-10

**Authors:** Sumati Hasani, Lyndsay E. A. Young, Warren Van Nort, Moumita Banerjee, Dylan R. Rivas, Jinhwan Kim, Xiaopeng Xiong, Ramon C. Sun, Matthew S. Gentry, Hiromi Sesaki, Tianyan Gao

**Affiliations:** 1 Department of Molecular and Cellular Biochemistry, University of Kentucky, Lexington, KY USA; 2 College of Agriculture, Food & Environment, University of Kentucky, Lexington, KY USA; 3 Markey Cancer Center, University of Kentucky, Lexington, KY 40536-0679 USA; 4 Department of Biochemistry and Molecular Biology, University of Florida, Gainesville, FL USA; 5 Department of Cell Biology, Johns Hopkins University School of Medicine, Baltimore, MD USA

**Keywords:** Metabolomics, Cancer metabolism

## Abstract

Metabolic reprogramming has been recognized as one of the major mechanisms that fuel tumor initiation and progression. Our previous studies demonstrate that activation of Drp1 promotes fatty acid oxidation and downstream Wnt signaling. Here we investigate the role of Drp1 in regulating glycogen metabolism in colon cancer. Knockdown of Drp1 decreases mitochondrial respiration without increasing glycolysis. Analysis of cellular metabolites reveals that the levels of glucose-6-phosphate, a precursor for glycogenesis, are significantly elevated whereas pyruvate and other TCA cycle metabolites remain unchanged in Drp1 knockdown cells. Additionally, silencing Drp1 activates AMPK to stimulate the expression glycogen synthase 1 (GYS1) mRNA and promote glycogen storage. Using 3D organoids from Apc^f/f^/Villin-Cre^ERT2^ models, we show that glycogen levels are elevated in tumor organoids upon genetic deletion of Drp1. Similarly, increased GYS1 expression and glycogen accumulation are detected in xenograft tumors derived from Drp1 knockdown colon cancer cells. Functionally, increased glycogen storage provides survival advantage to Drp1 knockdown cells. Co-targeting glycogen phosphorylase-mediated glycogenolysis sensitizes Drp1 knockdown cells to chemotherapy drug treatment. Taken together, our results suggest that Drp1-loss activates glucose uptake and glycogenesis as compensative metabolic pathways to promote cell survival. Combined inhibition of glycogen metabolism may enhance the efficacy of chemotherapeutic agents for colon cancer treatment.

## Introduction

Cancer cells are known to undergo metabolic reprogramming as a response to various stress conditions. Recent studies have focused on identifying mechanisms that allow cancer cells to acquire metabolic plasticity based on the type of nutrients available in the tumor microenvironment [1]. Increasing evidence suggests that mitochondria function as a key metabolic platform connecting the production of various metabolites with cell signaling and cell fate determination. In addition, the dynamic changes within the mitochondrial architecture have been shown to play an important role not only in supporting mitochondrial health and function but also cancer cell metabolism [2]. The term “mitochondrial dynamics” refers to a collection of mitochondrial movements, including continuous cycles of membrane fusion and fission and interactions with other organelles [3]. It has been shown that energy stress conditions can cause a shift in mitochondrial dynamics towards fusion and consequential changes in glucose metabolism, suggesting that mitochondrial architecture can play an important role in amending metabolic programs to sustain cell growth [4].

Elongated mitochondrial networks undergo fission to promote mitochondrial fragmentation, an essential checkpoint step required for cell division [3]. Mitochondrial fission is regulated by dynamin-related protein 1 (Drp1), a member of the dynamin family GTPases, that is cytosolically located and recruited to the outer mitochondrial membrane upon activation [5]. Drp1 activation downstream of oncogenic KRAS and BRAF signaling pathways has been shown to promote cellular metabolic reprogramming and tumor growth [6, 7]. Moreover, increased mitochondrial fragmentation events mediated by enhanced expression and activity of Drp1 have been identified as a ubiquitous signature in many cancer types [8–11], thus making Drp1 an attractive therapeutic target [12].

Although the functional interplay between mitochondrial dynamics and glycolysis has been extensively investigated [7, 13–15], it is less understood how Drp1 and mitochondrial fission regulate other glucose-dependent metabolic processes. Glycogenesis is a process in which glucose is converted into glycogen for the purpose of glucose storage. This high-density glucose polymer can undergo glycogenolysis, or the breakdown of glycogen into glucose, for energy production [16]. Glucose-6-phosphate (G6P) produced from glucose serves as a pre-cursor for both glycogenesis and glycolysis pathways. In glycogenesis, G6P primes the rate-limiting enzyme GYS1, to generate glycogen [17]. Glycogenolysis is regulated by glycogen phosphorylase, the key enzyme that breaks down α-1,4- and α-1,6-glycosidic bonds to form glucose-1-phosphate, which is then converted into usable glucose [18]. Recent studies have begun to establish the functional significance of glycogen in tumor initiation and progression. For example, hypoxic conditions are known to upregulate glycogenesis to support cell growth in breast cancer [19]. Intriguingly, liver-specific deletion of glycogen phosphorylase liver form (PYGL) results in increased glycogen storage and tumor development in mice [20]. Moreover, nuclear glycogen accumulation in non-small cell lung cancer promotes tumor growth in vivo by modulating histone acetylation [21].

In this study, we investigate the role of Drp1-mediated mitochondrial fission in regulating glycogen metabolism in colon cancer. Results from our study show that silencing Drp1 expression activates a compensatory metabolic pathway, in which increased glucose uptake is shuttled into the glycogenesis pathway resulting in glycogen accumulation in colon cancer cells. This increased glycogen storage functions as a survival mechanism in Drp1-deficient cells; and co-targeting glucogenolysis overcomes the pro-survival effect provided by glycogen. Taken together, our findings uncover a novel function of Drp1 in dictating the metabolic fate of glucose.

## Materials and methods

### Cell culture and reagents

Patient-derived colon cancer PT130 cells were established as previously described [22, 23]. SW480 cells were purchased from ATCC. Both cell lines were maintained in DMEM (high glucose) supplemented with 10% fetal bovine serum (FBS, Sigma-Aldrich) and 1% penicillin-streptomycin. For experiments analyzing glycogen related phenotypes, cells were cultured in DMEM containing 5 mM glucose (low glucose) supplemented with 10% FBS and 1% penicillin-streptomycin. The cell lines were authenticated using short tandem repeat (STR) DNA profiling and tested negative for mycoplasma contamination (Genetica). The following reagents were obtained from commercial sources: 1,4-dideoxy-1,4-imino-D-Arabinitol hydrochloride (DAB) was from Cayman Chemical (#20939), irinotecan hydrochloride and Compound C (AMPK inhibitor) were from MilliporeSigma (I1406 and 171260, respectively); control siRNA and siRNA targeting GYS1 was obtained from Santa Cruz Biotechnology. Stable control and Drp1 knockdown cells were generated using lentivirus-mediated RNAi as described previously [10]. The shRNA lentiviral plasmids were obtained from the Mission (MilliporeSigma). The targeting sequences for human Drp1 (DNM1L gene) are as the following: 5’-GCTACTTTACTCCAACTTATT-3’ (B3) and 5’-CGGTGGTGCTAGAATTTGTTA-3’ (B4).

### Seahorse extracellular flux analysis

The Seahorse XF96 extracellular flux analyzer (Agilent) was used to measure the mitochondrial and glycolytic activity of colon cancer cells as described previously [10, 23, 24]. Cells were seeded at the density of 3 × 10^4^ and 2.5 × 10^4^ cells per well for PT130 and SW480, respectively, in a XF96 plate approximately 16 h before the measurement. The mitochondrial and glycolysis stress tests were performed according to manufacturer’s protocol. The levels of basal, maximal and spear mitochondrial respiration were calculated based on OCR data obtained in the Mito Stress tests, whereas the levels of glycolysis, glycolytic capacity and glycolytic reserve were calculated based on ECAR data obtained in the Glycolysis Stress tests using Seahorse Wave software (Agilent). The number of cells per well was determined using Seahorse XF Imaging and Cell Counting software and all OCR and ECAR measurements were normalized to cell numbers in each well.

### Glucose uptake assays

Sh-Ctrl and sh-Drp1 PT130 or SW480 cells were seeded at 1.0 × 10^4^ cells/well in a 96 well assay plate and were cultured for 48 h in DMEM (high glucose) supplemented with 10% FBS and 1% penicillin/streptomycin. Cells were then incubated with 400 nM 2-NBDG (2-(N-(7-Nitrobenz-2-oxa-1,3-diazol-4-yl)Amino)-2-Deoxyglucose) and 2 μM Hoechst 33342 Solution (Thermofisher) for 1 h. Cells were washed with PBS twice and levels of fluorescence labeling were read using the Varioskan Lux microplate reader. Levels of 2-NBDG fluorescence were normalized to cell density as determined by Hoechst staining.

### Immunofluorescence (IF) imaging

To examine the levels of glycogen and Drp1 expression, sh-Ctrl and sh-Drp1 PT130 and SW480 cells were seeded on glass coverslips and cultured in low glucose DMEM supplemented with 10% FBS for 24 h. Cells were fixed with 4% paraformaldehyde and co-stained with antibodies against Drp1 and glycogen (IV58B6 [25]). All images were captured using the same acquisition settings among experimental groups. To determine the effect of AMPK inhibition on glycogen accumulation, cells were treated with Compound C in low glucose medium for 24 h (10 μM and 20 μM for PT130 and SW480 cells, respectively). The concentration of Compound C was selected based on the status of phospho-AMPK inhibition. For experiments targeting GYS1, cells transfected control or GYS1 targeting siRNA were switched to low glucose media 48 h post transfection and cultured for additional 24 h. Images were taken using Nikon A1^+^ confocal microscope and the mean fluorescence intensity was determined using the ImageJ analysis toolset [26]. Twenty cells randomly selected from at least ten different imaging fields for each group were used for quantification.

### Western blot analysis

Total protein lysates were prepared by incubating cells or tumor tissues in lysis buffer [50 mM Na_2_HPO_4_, pH 7.4, 1 mM sodium pyrophosphate, 20 mM NaF, 2 mM EDTA, 2 mM EGTA, 1% Triton X-100, 1 mM DTT and protease inhibitor cocktail (MilliporeSigma)] for 5-10 min on ice and the detergent-insoluble debris was removed after centrifugation for 5 min at 16,000*g* at 4 °C [27–30]. Equal amounts of total protein lysates were resolved by SDS-PAGE and subjected to western blot analysis. The following antibodies were used: from Cell Signaling, Drp1 (#5391), GYS1 (#3893), p-ACC (#3661), ACC (#3662), p-AMPK (#2531), AMPK (#2532); and from MilliporeSigma, β-actin (A1978).

### Quantitative RT-PCR (RT-qPCR)

To determine the relative gene expression, total RNAs were extracted from cells or tumor tissues using the PureLink^TM^ RNA Mini Kit (ThermoFisher). The reverse transcription and quantitative PCR reactions were carried out as described previously [22–24]. Primers used in this study are shown in the Supplemental Materials. All values were normalized to the level of β-actin.

### Metabolite extraction and analysis

Sh-Control and sh-Drp1-B3 PT130 cells were cultured in low glucose DMEM containing 10% FBS and 1% penicillin/streptomycin for 18 h. Cells were washed with PBS twice, treated with 50% methanol and incubated on ice for 10 min. Cells lysates were collected and L-norvaline was added as internal control. Polar metabolites were extracted and processed as described previously [21, 31]. For GC-MS analysis, an Agilent 7800B gas–chromatography coupled to a 7010A triple quadrupole mass spectrometry detector equipped with a high-efficiency source was used. Data analysis was performed using Aglient Masshunter software. Polar metabolites were normalized to the total biomass fraction consisting of amino acids found in the protein GC-MS quantitation. Data were normalized by Log and auto scaling for comparison.

### Cell proliferation in tumor organoids

Drp1^f/f^ mice were previously described and maintained on a C57BL/6 background [32]. To produce animals used in this study, Drp1^f/f^ mice were crossed with Apc^f/f^ [33] and Villin-Cre^ERT2^ mice to create Apc^f/f^/Vil-Cre^ERT2^ (Apc) and Apc^f/f^/Drp1^f/f^/Vil-Cre^ERT2^ (Apc/Drp1-KO) mice. Drp1^f/f^ mice were generously provided by Dr. Hiroshi Sasaki and the other two mouse strains were obtained from The Jackson Laboratory (Bar Harbor, ME). The mice were then inter-crossed to produce mice used for isolating intestinal organoids. Both male and female mice of 8–10 weeks were included in each group. To induce deletion of Drp1 and Apc genes, mice were injected with a single-dose of tamoxifen (0.5 mg/mouse) and intestinal tissues were extracted at 1-week post-injection. To propagate Apc-driven tumor organoids, intestinal crypts were isolated as described previously [24] and maintained in tumor organoid media (Advanced DMEM/F12 supplemented with 1×Glutamax, 1×HEPES, 1×N-2, 1×B-27, 1 mM NAcetyl-l-cysteine, 1% penicillin/streptomycin and 50 ng/ml EGF). To determine cell proliferation and growth, Apc and Apc/Drp1-KO organoids were dissociated into single cells using TrypLE (Thermofisher). Equal numbers of cells were subseeded into 12-well plate (10,000 cells/well) in 50% Matrigel and cultured in tumor organoid media for 3–4 days.

To detect proliferating cells in tumor organoids, Apc and Apc/Drp1-KO tumor organoids grown in 3D were incubated with 5-ethynyl-2′-deoxyuridine (EdU) for 2 h prior to fixation. The EdU positive cells were stained using Click-iT EdU Alexa Fluor 594 Imaging Kit (Thermofisher). The organoids were washed with PBS and resuspended in DAPI-mounting media. Images were taken using a Nikon A1^+^ confocal microscope. Additionally, the CellTiter-Glow 3D viability assays (Promega) were carried out in tumor organoids to quantitatively measure cell growth.

### Glycogen measurement in xenograft tumors

Xenograft tumors derived from sh-Ctrl and sh-Drp1-B3 PT130 cells were generated as previously described [10]. To determine levels of glycogen accumulation, sections of tumor tissues were subjected to immunohistological staining using the anti-glycogen antibody (IV58B6). The percentage of tumor cells with positive staining of glycogen was quantified using the HALO image analysis platform (Indica Labs).

### Cell survival assays

To determine the effect of Drp1 knockdown on cell survival under glucose starvation or drug treatment conditions, sh-Ctrl and sh-Drp1 PT130 or SW480 cells were seeded at 1 × 10^4^ cells/well in a 96-well plate and were cultured in regular growth media for 24 h. For glucose starvation, cells were cultured in glucose-free media (XF DMEM medium supplemented with 2 mM glutamine, 10% FBS and 1% penicillin/streptomycin) for 48 h (for PT130) or 72 h (for SW480). Due to intrinsic differences in their proliferation rates, the two cell lines were subjected to the inhibitor treatment for different amount of time in order to reach similar levels of inhibition. For irinotecan and DAB treatment assays, cells were cultured in regular growth media for 24 h and subsequently treated with irinotecan (1 μM), DAB (15 μM) or combinations of irinotecan and DAB for addition 48 h (PT130) or 72 h (SW480). At the end of the treatment, cells were labeled with Hoechst 33342 Solution (2 μM) for 1 h. After washing with PBS, the levels of Hoechst fluorescence staining were determined using the Varioskan Lux microplate reader.

### Statistical and bioinformatics analysis

Data from at least three independent experiments are expressed as means with SD as indicated in figure legends. Statistical analysis was performed using the student *t*-test for pairwise comparisons and one-way ANOVA for multiple comparisons. For the quantitative protein and mRNA expression analysis in xenograft tumors, average of four tumors in the control and four tumors in the sh-Drp1 group were used. For the Gene Set Enrichment Analysis (GSEA), RNA sequencing data were obtained from the TCGA COAD study. Correlations between expressions of Drp1 (gene name: *DNM1L*) and other genes were quantified by Spearman’s correlation coefficient. The genes were then ordered from highest to lowest based on the correlation coefficient. This ranked list was inputted into the GSEA Desktop Application [34] to identify pathways that are associated with Drp1 expression.

## Results

### Knockdown of Drp1 alters glucose metabolism

**Fig. 1:**
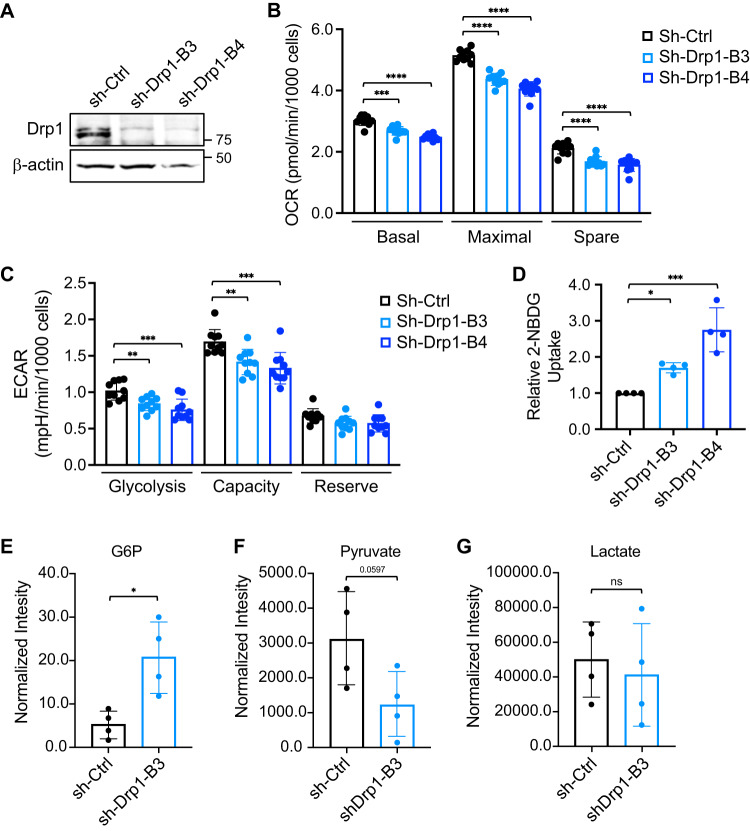
Knockdown of Drp1 alters glucose metabolism. **A** Cell lysates of control (sh-Ctrl) and two Drp1 knockdown (sh-Drp1-B3 and sh-Drp1-B4) PT130 cells were analyzed for the expression of Drp1 and β-actin using western blot. **B** Control and Drp1 knockdown PT130 cells were subjected to Mito Stress tests using Seahorse XF96 Extracellular Flux Analyzer as described in Materials and methods. The OCR measurements associated with basal, maximal, and spare capacity of mitochondrial respiration were calculated by normalizing to total cell numbers. Data represent the mean ± SD (*n* = 10, ***p < 0.001 and ****p < 0.0001). **C** Control and Drp1 knockdown PT130 cells were subjected to Glycolysis Stress tests using Seahorse XF96 Extracellular Flux Analyzer. The ECAR measurements associated with glycolysis, glycolytic capacity, and glycolytic reserve were calculated by normalizing to total cell numbers. Data represent the mean ± SD (*n* = 10, **p < 0.01 and ***p < 0.001). **D** Control and Drp1 knockdown PT130 cells were incubated with 2-NBDG and Hoechst in low glucose media for 1 h. Relative glucose uptake was calculated by normalizing fluorescence signals detected from 2-NBDG to Hoechst. Data were presented as mean ± SD (*n* = 4, *p < 0.05 and ***p < 0.001). **E**–**G** Control and Drp1 knockdown PT130 cells were cultured in low glucose media for 18 h. Polar metabolites were extracted and analyzed using GC-MS. The levels of metabolites, including G6P (**E**), pyruvate (**F**), and lactate (**G**), in the glycolytic pathway are shown. Data were presented as mean ± SD (*n* = 4, *p < 0.05).

Since Drp1 plays a key role in maintaining the integrity of mitochondrial networks, we first determined the effect of silencing Drp1 on altering mitochondrial activity. Stable Drp1 knockdown lines were generated using lentivirus-mediated RNAi in PT130 (Fig. 1A) and SW480 cells (Supplementary Fig. S1A). We have shown previously that silencing Drp1 in both cell lines results in mitochondrial elongation confirming the function of Drp1 as a fission regulator [10]. Control and Drp1 knockdown cells were subjected to Mito Stress tests to measure mitochondrial respiration using the Seahorse Extracellular Flux Analyzer. Quantitative results from the Seahorse analysis indicated that oxygen consumption rates (OCRs) associated basal, maximal, and spare respiration of mitochondria were significantly decreased in Drp1 knockdown PT130 cells (Fig. 1B). Similarly, knockdown of Drp1 modestly decreased maximal and spare respiration of mitochondria in SW480 cells (Supplementary Fig. 1B). Since cancer cells are known to upregulate glycolysis when the mitochondrial activity is inhibited, we next compared glycolysis phenotypes in control and Drp1 knockdown cells using the Seahorse Glycolysis Stress tests. Intriguingly, we found that the extracellular acidification rate (ECAR) associated with glycolysis and glycolytic capacity was decreased whereas the reserved capacity remained unchanged in Drp1 knockdown PT130 (Fig. 1C) and SW480 cells (Supplementary Fig. S1C), suggesting that inhibition of mitochondrial fission also blocks the compensatory upregulation of glycolysis typically observed in cells with impaired mitochondrial respiration. Despite decreased glycolytic activity observed in Drp1 knockdown cells, the rate of glucose uptake was increased compared to sh-Ctrl cells in both PT130 (Fig. 1D) and SW480 (Supplementary Fig. S1D).

To better understand the downstream effect of this paradoxical coupling of decreased glycolysis with increased glucose uptake, we measured essential cellular metabolites in PT130 control and Drp1 knockdown cells using GC-MS. Cells were cultured in low glucose media to mimic physiologically relevant conditions. Consistent with increased glucose uptake, we observed a significant increase in G6P (Fig. 1E), the first metabolite derived from glucose, in Drp1 knockdown cells. However, silencing Drp1 led to decreased cellular pyruvate levels (Fig. 1F) whereas the lactate production remained unchanged (Fig. 1G). Taken together with our findings from the Seahorse analysis in which both mitochondrial respiration and glycolytic potential are decreased in Drp1 knockdown cells, these data suggest that increased glucose uptake in response to Drp1-loss may adopt a different metabolic fate than feeding into glycolysis and downstream lactate fermentation pathway.

### Knockdown of Drp1 promotes glycogen accumulation

**Fig. 2:**
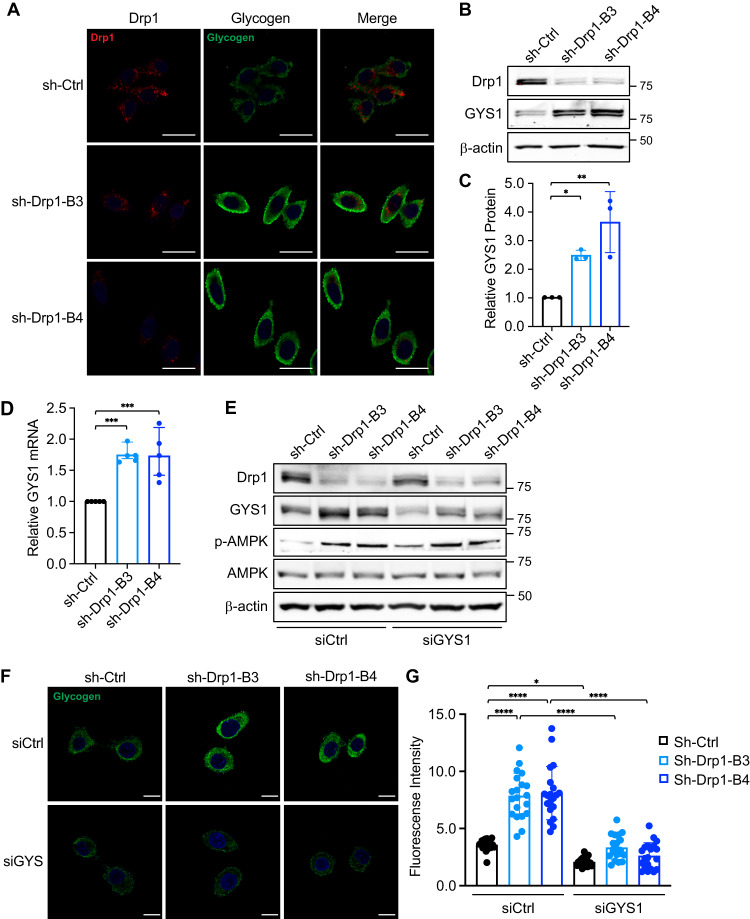
Knockdown of Drp1 promotes glycogen accumulation. **A** Representative confocal images of control (sh-Ctrl) and Drp1 knockdown (sh-Drp1-B3 and sh-Drp1-B4) PT130 cells that were stained with antibodies against Drp1 (red) and glycogen (green). Scale Bar, 20 μm. **B** Cell lysates from sh-Ctrl and sh-Drp1 PT130 cells were analyzed for the expression of Drp1, GYS1 and β-actin using western blot. **C** Representative western blots as shown in (**B**) were quantified to determine the relative GYS1 levels by normalizing GYS1 to β-actin. Data were presented as mean ± SD (*n* = 3, *p < 0.05 and **p < 0.01). **D** sh-Ctrl and sh-Drp1 PT130 cells cultured in low glucose media were analyzed for the expression of GYS1 mRNA using RT-qPCR. Data were presented as mean ± SD (*n* = 3, ***p < 0.001). **E** Sh-Ctrl and sh-Drp1 PT130 cells were transfected with non-targeting (siCtrl) or GYS1-specific siRNA to silence GYS1 expression. Cell lysates were analyzed for the expression of Drp1, GYS1, *p*-AMPK, AMPK, and β-actin using western blot. **F** Sh-Ctrl and sh-Drp1 PT130 cells were transfected as described in (**E**). Representative confocal images were obtained from cells stained with the anti-glycogen antibody. Scale Bar, 10 μm. **G** The relative fluorescence intensity of glycogen staining was quantified using ImageJ fluorescence analyzer. Data were presented as mean ± SD (*n* = 20, *p < 0.05 and ****p < 0.0001).

Since G6P functions as a metabolic pre-cursor that can feed into both glycolysis and glycogenesis pathways [35], we next examined if knockdown of Drp1 promotes the conversion of glucose and G6P into glycogen via glycogenesis in colon cancer cells. To detect the endogenous glycogen expression, we performed immunofluorescence staining in control and Drp1 knockdown cells using the anti-glycogen antibody IV58B6. Cells were co-stained with the Drp1 antibody to confirm the reduced expression of Drp1 in knockdown cells. The basal levels of glycogen were relatively low in PT130 sh-Ctrl cells where Drp1 expression was readily detected (Fig. 2A). Interestingly, we observed a marked increase in cytosolic glycogen accumulation in Drp1 knockdown PT130 cells as compared to control cells (Fig. 2A). To determine the underlying mechanisms contributing to increased glycogen levels, we analyzed the expression of GYS1, a key enzyme needed to assemble G6P into glycogen [36]. Indeed, the expression levels of GYS1 protein and mRNA were significantly increased in Drp1 knockdown PT130 cells (Fig. 2B–D). Similar increases in glycogen accumulation and GYS1 expression were observed in Drp1 knockdown SW480 cells (Supplementary Fig. S2A–D).

To confirm the requirement of GYS1 for glycogen accumulation, we silenced GYS1 expression using siRNA targeting GYS1 in control and Drp1 knockdown cells (Fig. 2E). We then visualized the effect of GYS1 depletion on glycogen accumulation using IF staining (Fig. 2F). Consistently, results from the quantitative analysis showed that while knockdown of Drp1 significantly increased the levels of glycogen, GYS1 depletion abolished the effect of Drp1-loss on promoting glycogen accumulation (Fig. 2G). In addition, we found that knockdown of Drp1 induced AMPK activation (Fig. 2E), likely in response to the decrease in mitochondrial respiration as shown in the Seahorse analysis. Collectively, these results indicate that GYS1 is transcriptionally upregulated in Drp1 knockdown cells and increased expression of GYS1 promotes glycogen accumulation.

### Disruption of mitochondrial fission activates AMPK to promote GYS1 expression in Drp1 knockdown cells

**Fig. 3:**
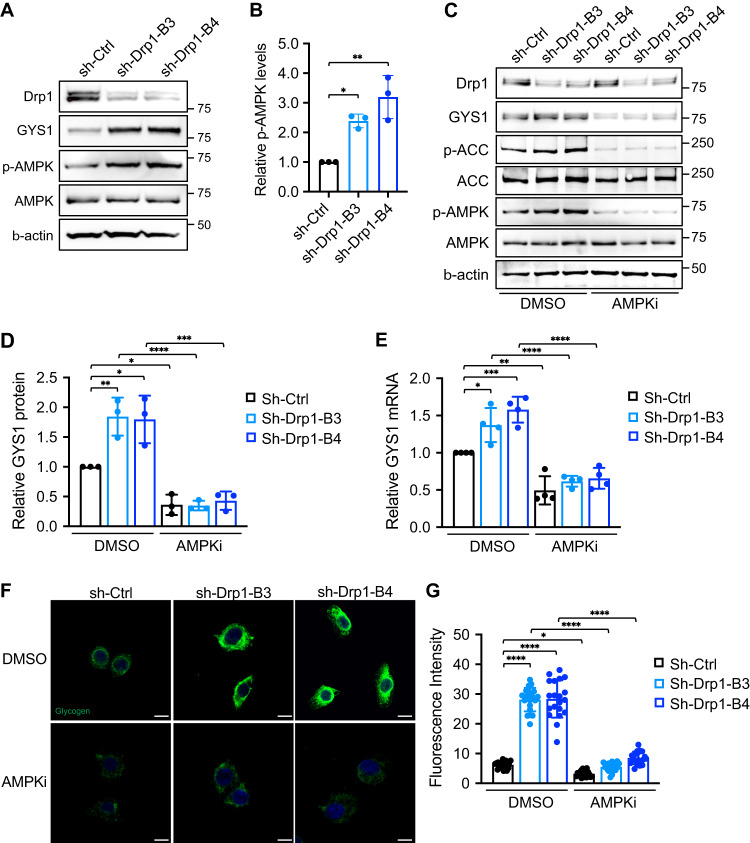
Increased GYS1 expression in Drp1 knockdown cells is transcriptionally regulated by AMPK. **A** Cell lysates of sh-Ctrl, sh-Drp1 PT130 cells were analyzed for the expression of Drp1, GYS1, phospho-AMPK (p-AMPK), total AMPK and β-actin using western blot. **B** Representative western blot as shown in (**A**) were quantified to determine the relative p-AMPK levels by normalizing p-AMPK to total AMPK. Data were presented as mean ± SD (*n* = 3, *p < 0.05 and **p < 0.01). **C** Sh-Ctrl and sh-Drp1 PT130 cells were treated with DMSO or AMPK inhibitor compound C (AMPKi, 10 μM) for 24 h in low glucose media. Cell lysates were analyzed for the expression of Drp1, GYS1, p-AMPK, phospho-ACC (p-ACC), total ACC, total AMPK, and β-actin using western blot. **D** Representative western blots as shown in (**C**) were quantified to determine the relative GYS1 levels by normalizing GYS1 to β-actin. Data were presented as mean ± SD (*n* = 3, *p < 0.05, **p < 0.01, ***p < 0.001 and ****p < 0.0001). **E** The relative expression of GYS1 mRNA was determined using RT-qPCR in sh-Ctrl and sh-Drp1 PT130 cells treated with DMSO or AMPKi. Data were presented as mean ± SD (*n* = 3, *p < 0.05, **p < 0.01, ***p < 0.001 and ****p < 0.0001). **F** Sh-Ctrl and sh-Drp1 PT130 cells were treated with DMSO or AMPKi in low glucose media for 24 h. Representative confocal images were obtained from cells stained with the anti-glycogen antibody. Scale Bar, 10 μm. **G** The relative fluorescence intensity of glycogen staining was quantified using ImageJ fluorescence analyzer. Data were presented as mean ± SD (*n* = 20, *p < 0.05 and ****p < 0.0001).

Previous studies have linked the activation status of AMPK to glycogenesis [37]. To further investigate the molecular mechanisms underlying increased GYS1 expression and subsequential glycogen accumulation in Drp1 knockdown cells, we determined if upregulation of AMPK activity regulates GYS1 expression in Drp1 knockdown cells. We first confirmed that knockdown of Drp1 knockdown significantly increased AMPK phosphorylation, which coincided with elevated GYS1 expression in PT130 (Fig. 3A, B) and SW480 cells (Supplementary Fig 3A, B). To determine if AMPK activation increases GYS1 transcription, we treated control and Drp1 knockdown PT130 (Fig. 3C) and SW480 cells (Supplementary Fig. S3C) with AMPK inhibitor (AMPKi), Compound C. We showed that blocking AMPK activity significantly decreased GYS1 protein expression in both control and Drp1 knockdown PT130 (Fig. 3D). The phosphorylation of ACC was decreased in AMPKi treated cells confirming AMPK inhibition. Additionally, we analyzed the expression of GYS1 mRNA using RT-qPCR in control and Drp1 knockdown PT130 cells treated with AMPKi (Fig. 3E). Quantitative results indicated that both basal and Drp1-loss induced GYS1 upregulation were diminished upon AMPK inhibition, confirming that activation of AMPK in Drp1 knockdown cells is responsible for increased GYS1 mRNA expression. Similarly, we demonstrated that inhibition of AMPK in control and Drp1 knockdown SW480 cells effectively reduced the expression of GYS1 at both the protein and mRNA levels (Supplementary Fig. S3E).

To better understand the implications of enhanced AMPK activity in the context of glycogen accumulation, we examined the changes in glycogen levels in control and Drp1 knockdown cells when treated with AMPKi using IF staining. Indeed, AMPKi treatment significantly reduced glycogen accumulation in both control and Drp1 knockdown PT130 (Fig. 3F, G) and SW480 cells (Supplementary Fig. S3F, G). Taken together, our results suggested that the glycogen accumulation phenotype observed in Drp1 knockdown cells is regulated through the AMPK/GYS1 signaling axis.

### Deletion of Drp1 enhances glycogen accumulation in Apc-derived tumor organoids

**Fig. 4:**
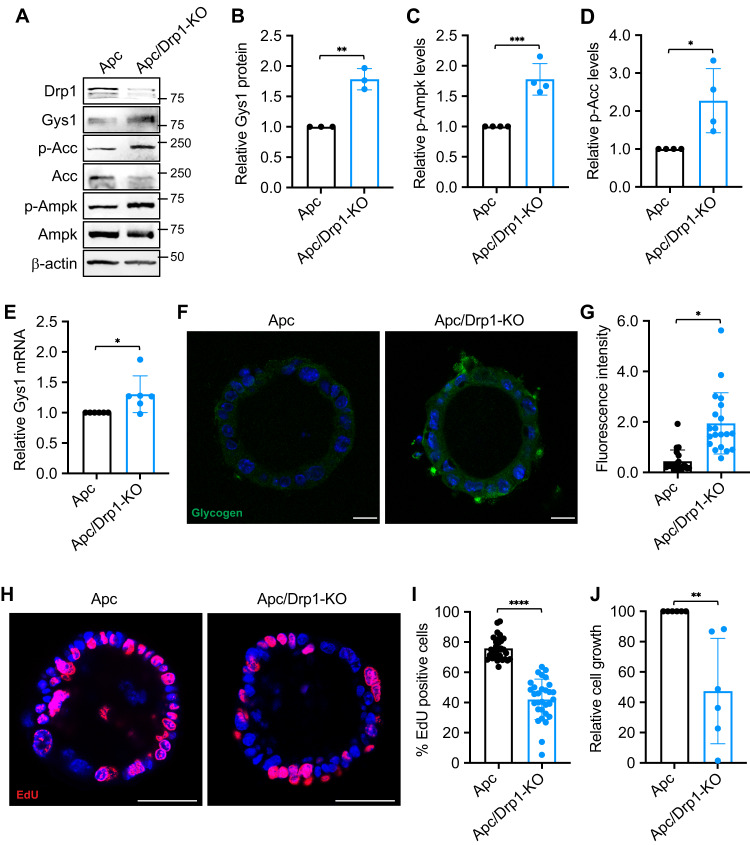
Depletion of Drp1 in APC-derived tumor organoids increases glycogen storage. **A** Apc^f/f^/Vil-Cre^ERT2^ (Apc) and Apc^f/f^/Drp1^f/f^/Vil-Cre^ERT2^ (Apc/Drp1-KO) organoids were cultured in 3D Matrigel for 4 days. Cell lysates from organoids were analyzed for the levels of Drp1, Gys1, p-Acc, total Acc, p-Ampk, total Ampk and β-actin using western blot. **B**–**D** Representative western blots as shown in (**A**) were quantified to determine the expression Gys1, p-Ampk and p-Acc. The relative expression levels of Gys1 were obtained by normalizing Gys1 to β-actin (**B**), whereas the phosphorylation levels of Ampk and Acc were determined by normalizing p-Ampk and p-Acc to that of total AMPK and Acc, respectively (**C**, **D**). Data were presented as mean ± SD (*n* = 4, *p < 0.05, **p < 0.01 and ***p < 0.001). **E** The relative expression of Gys1 mRNA was determined using RT-qPCR in Apc and Apc/Drp1-KO organoids. Data were presented as mean ± SD (*n* = 6, *p < 0.05). **F** Representative confocal images were obtained from Apc and Apc/Drp1-KO organoids stained with the anti-glycogen antibody. Scale Bar, 20 μm. **G** The relative fluorescence intensity of the glycogen staining was quantified using ImageJ fluorescence analyzer. Data were presented as mean ± SD (*n* = 20, *p < 0.05). **H** Apc and Apc/Drp1-KO organoids cultured in 3D Matrigel for 3 days were labeled with EdU to mark proliferating cells. EdU positive cells were visualized using Click-it EdU Alexa 594. **I** The percentage of EdU positive cells were quantified in Apc and Apc/Drp1-KO organoids. Data were presented as mean ± SD (*n* = 30, ****p < 0.0001). **J** The relative cell growth of Apc and Apc/Drp1-KO organoids were quantified using the CellTiter-Glo 3D Luminescent Cell Viability Assay Kit. The luminescence signals were normalized to the number of organoids formed. Data were presented as mean ± SD (*n* = 6, **p < 0.01).

To further evaluate the effect of Drp1 deletion on glycogen metabolism, we generated Apc and Apc/Drp1-KO tumor organoids. Since intestinal specific knockout of both Apc alleles results in lethality within 7–10 days as previously reported [33], we collected intestinal tissues from single or double knockout mice 7-day after tamoxifen injection and selected Apc-derived tumor organoids for in vitro experiments in 3D. To determine the expression of GYS1, Apc and Apc/Drp1-KO tumor organoids were cultured in 3D Matrigel for 1 week and collected for western blot analysis (Fig. 4A). Consistent with our results obtained in colon cancer cell lines, Apc/Drp1-KO organoids exhibited a significant increase in GYS1 protein expression (Fig. 4B) which coincided with an increase in AMPK and ACC phosphorylation (Fig. 4C, D). Additionally, we assessed GYS1 mRNA levels using RT-qPCR and verified that GYS1 gene expression was upregulated in Apc/Drp1-KO compared to Apc tumor organoids (Fig. 4E).

Furthermore, we monitored glycogen levels in 3D tumor organoids by IF staining using the anti-glycogen antibody. As shown in Fig. 4F, increased levels of glycogen staining were detected in Apc/Drp1-KO organoids. Quantitative analysis confirmed that the deletion of Drp1 significantly increased glycogen accumulation (Fig. 4G). In addition, we determined the rate of cell proliferation in Apc and Apc/Drp1-KO tumor organoids. Cells grown in 3D were labeled with EdU and the number of EdU positive cells were quantified. Interestingly, genetic deletion of Drp1 significantly decreased EdU positive cell population in Apc/Drp1-KO tumor organoids as compared to Apc control (Fig. 4H, I). Consistently, the expression of cell proliferation marker CCND1 was decreased in Apc/Drp1-KO tumor organoids as determined by RT-qPCR analysis (Supplementary Fig. S4A). Moreover, results from Cell TiterGlo Assay revealed that the total growth of Apc/Drp1-KO tumor organoids was significantly reduced as well (Fig. 4J). However, genetic ablation of Drp1 did not alter the number of colonies formed in 3D (Supplementary Fig. S4B). Taken together, our results showed that deletion of Drp1 in APC-derived tumor organoids promotes the expression of GYS1 and glycogen accumulation. Intriguingly, while Drp1-loss decreases cell proliferation, the ability of tumor organoids to form new colonies in vitro was not affected, suggesting that glycogen accumulation downstream of Drp1-loss may not be involved in tumor initiation in the Apc-driven tumor models.

### Depletion of Drp1 increases glycogen storage in xenograft tumors

**Fig. 5:**
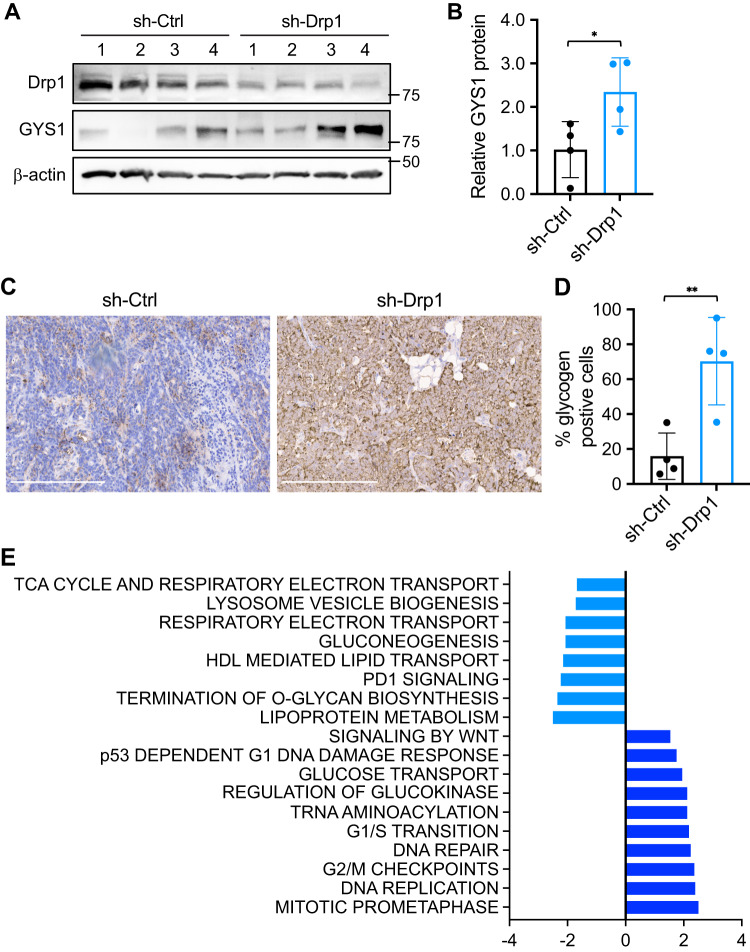
Depletion of Drp1 increases glycogen storage in vivo. **A** Sh-Ctrl and sh-Drp1 PT130 cells were injected subcutaneously into NSG mice. Tumor tissues from both groups were analyzed for the expression of Drp1, GYS1 and β-actin using western blot. **B** The relative GYS1 levels in sh-Ctrl and sh-Drp1 tumors detected by western blot as shown in (**A**) were quantified by normalizing GYS1 to β-actin. Data were presented as mean ± SD (*n* = 4, *p < 0.05). **C** Representative images were obtained from sh-Ctrl and sh-Drp1 tumor sections stained with the anti-glycogen antibody. Scale bar, 200 μm. **D** The percentage of glycogen positive cells were quantified in tumors from four mice of each group using the HALO software. Data were presented as mean ± SD (*n* = 4, **p < 0.01). **E** TCGA COAD RNA-seq dataset was first used to identify genes that have positive or negative correlations with Drp1 (gene name: DNM1L) expression. The GSEA was then performed to determine if DNM1L expression is associated with gene sets in the REACTOME collection. The name of the gene sets and the corresponding normalized enrichment score (NES) and false discovery rate (FDR) are listed in the graph (the cutoff for significance is set for FDR < 0.05).

We have previously shown that knockdown of Drp1 in colon cancer cells reduces xenograft tumor growth in vivo [10]. Here we utilized tumor tissues collected from control and Drp1 knockdown PT130 cells to determine if Drp1-loss induces glycogen accumulation in vivo. First, we found that knockdown of Drp1 significantly increased the GYS1 protein expression in xenograft tumors (Fig. 5A, B). Next, tumor tissues were subjected to IHC staining using the anti-glycogen antibody and the percentage of cells with positive staining was quantified using HALO imaging analysis. We found that the levels of glycogen accumulation were markedly enhanced in sh-Drp1 tumors as compared to sh-Ctrl (Fig. 5C, D). Together, these data confirmed that Drp1-loss promotes glycogen storage in vivo.

In addition, we mined the TCGA COAD RNA-seq dataset to identify candidate genes positively and negatively correlated with Drp1 (gene name: *DNM1L)* expression in colon cancer patients. Gene Set Enrichment Analysis (GSEA) was performed on the REACTOME collection of genes and candidates associated with *DNM1L* were identified and categorized into various pathways (Fig. 5E). Consistent with previously characterized functions of Drp1 in cell cycle, *DNM1L* gene expression is associated with cell cycle transition and checkpoints. Interestingly, *DNM1L* was also found to be associated with genes in mitochondrial respiratory pathways such as gluconeogenesis, TCA cycle and electron transport chain, regulation of glucokinase, and glucose transport pathways, confirming that Drp1 is functionally connected with glucose metabolism to regulate downstream metabolic programs in human colon cancer patients.

### Increased glycogen storage functions as a survival mechanism in Drp1 knockdown cells

**Fig. 6:**
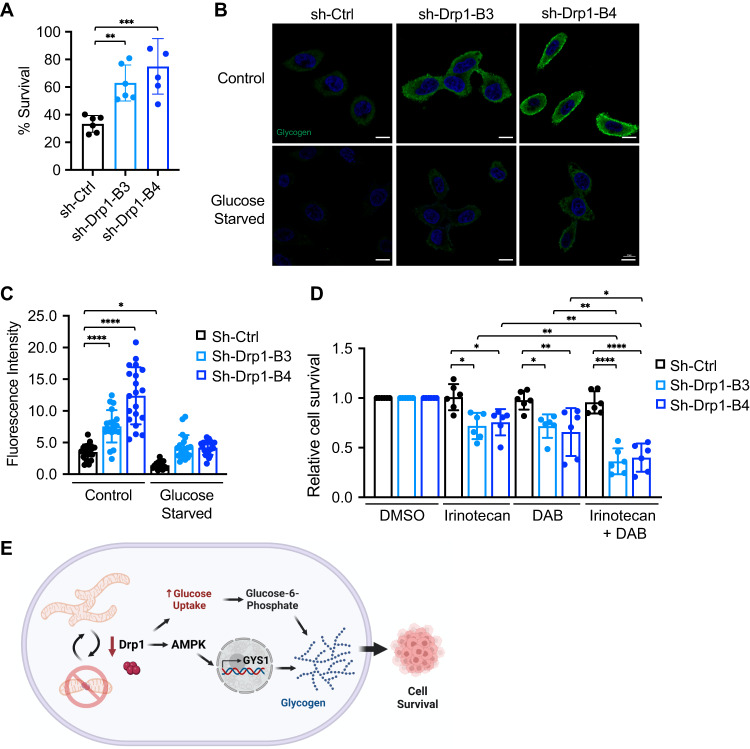
Increased glycogen storage functions as a survival mechanism in Drp1 knockdown cells. **A** Sh-Ctrl and sh-Drp1 PT130 cells were cultured in regular growth media or glucose-free medium for 48 h. The percentage of cell survival were obtained by normalizing the number of cells survived in glucose-free media to that of regular growth media. Data were presented as mean ± SD (*n* = 6, **p < 0.01 and ***p < 0.001). **B** Sh-Ctrl and sh-Drp1 PT130 cells were cultured in low glucose or glucose-free media for 24 h. Representative confocal images were obtained from cells stained with the anti-glycogen antibody. Scale Bar, 10 μm. **C** The relative fluorescence intensity of glycogen staining was quantified using ImageJ fluorescence analyzer. Data were presented as mean ± SD (*n* = 20, *p < 0.05, **p < 0.01 and ****p < 0.0001). **D** Sh-Ctrl and sh-Drp1 PT130 cells were treated with irinotecan, DAB or combinations of both agents for 48 h. Cells treated with DMSO were used as control. The relative of cell survival were obtained by normalizing to cells treated with DMSO. Data were presented as mean ± SD (*n* = 6, *p < 0.05, **p < 0.01, ***p < 0.001 and ****p < 0.0001). **E** Results from our study demonstrate that disruption of mitochondrial dynamics as a consequence of silencing Drp1 increases glucose uptake and AMPK-dependent transcriptional activation of GYS1. Subsequently, GYS1-mediated glycogen accumulation serves as a compensatory survival mechanism to protect colon cancer cells from glucose starvation and chemotherapy treatment. Thus, co-targeting glycogenolysis may provide a novel strategy to sensitize Drp1 knockdown cells to chemotherapy.

To better understand the functional significance of glycogen accumulation in Drp1 knockdown cells, we tested the hypothesis that increased glycogen expression provides a survival advantage to help cancer cells cope with stress conditions. To test this, we first subjected control and Drp1 knockdown cells to the glucose starvation condition. Intriguingly, Drp1 knockdown PT130 (Fig. 6A) and SW480 (Supplementary Fig. S5A) cells survived better when cultured in glucose-free media as compared to control cells. We reasoned that increased glycogen levels can provide higher levels of glucose through glycogenolysis (glycogen breakdown) to support the survival of Drp1 knockdown cells when glucose availability becomes limited. We next monitored changes in glycogen levels under glucose starvation conditions by staining control and Drp1 knockdown cells with the anti-glycogen antibody (Fig. 6B). Indeed, glucose deprivation significantly reduced glycogen levels in both control and Drp1 knockdown PT130 (Fig. 6B, C) and SW480 (Supplementary Fig. S5B, C), confirming that glycogen storage in Drp1 knockdown cells serves as a glucose reserve to sustain cell survival.

To further determine the contribution of glycogen in cell survival, we tested if inhibiting glycogenolysis would diminish the survival advantage provided by increased glycogen storage in Drp1 knockdown cells. As glycogen phosphorylase is the rate-limiting enzyme that controls the breakdown of glycogen, we examined the effect of inhibiting glycogen phosphorylase on cell survival in control and Drp1 knockdown PT130 cells. To this end, cells were treated with a potent glycogen phosphorylase inhibitor, DAB [38], alone or in combination with irinotecan, a clinically relevant chemotherapy drug in colon cancer [39, 40]. While the concentrations of DAB and irinotecan used in our experiments did not induce significant cell death in control cells, Drp1 knockdown cells were more sensitive to either inhibitor treatment alone as shown by reduced survival compared to control cells (Fig. 6D). Importantly, the combination of irinotecan and DAB further decreased cell survival in Drp1 knockdown cells but not in control cells (Fig. 6D). Similar results were obtained in SW480 control and Drp1 knockdown cells (Supplementary Fig. S5D). Collectively, our data suggest that inhibition of glycogen conversion into glucose in Drp1 knockdown cells can further enhance the potency of chemotherapeutic drug.

In summary, our study highlights that Drp1 knockdown cells can employ compensatory metabolic mechanisms to enhance glycogen synthesis as a means of survival. Disruption of mitochondrial dynamics results in increased glucose uptake, activation of AMPK, and subsequent upregulation of GYS1 expression. Increased levels of G6P are ushered into the glycogenesis pathway to fuel glycogen production. Functionally, upregulation of cellular glycogen storage can be utilized by cancer cells to promote survival under stress conditions (Fig. 6E).

## Discussion

Mitochondria are dynamic organelles that undergo continuous changes in their architectures to support various functionalities [41]. Dysregulation of mitochondrial homeostasis has been identified as one of the hallmarks for cancer progression. Cancer cells are known to acquire ability to alter their mitochondrial morphology to accommodate increasing energy requirements [2]. Specifically, hyperactivation of Drp1 has been identified to not only increase mitochondrial scission, but also shift metabolic programs based on nutrient availability and exogenous factors within the tumor microenvironment [42]. In this study, we show that Drp1 depletion reduces mitochondrial respiration when glucose is used as the metabolic substrate in colon cancer cells. Although this downregulation of mitochondrial activity stimulates AMPK activation and increases glucose uptake, the glycolytic function is paradoxically decreased in Drp1 knockdown cells. Interestingly, we find that Drp1 knockdown cells utilize an alternative metabolic program in that glucose is converted into G6P and used for glycogen production. Mechanistically, increased AMPK activation promotes GYS1 expression to facilitate glycogen synthesis in multiple model systems, including Drp1 knockdown colon cell lines, Drp1 knockout Apc-driven tumor organoids and xenograft tumor tissues derived from Drp1 knockdown cells. In addition, we demonstrate that increased glycogen accumulation functions as an energy storage to support the survival of cancer cells with mitochondrial deficiency. As Drp1-targeted cancer therapies are being explored as potential treatment options to inhibit cancer progression and survival [43], our findings indicate that blocking glycogenolysis may provide additional benefits to circumvent glycogen-mediated pro-survival effect.

The role of AMPK in regulating the balance of glycogen synthesis and glycogenolysis has been intensively investigated in previous studies [44]. It has been shown that AMPK directly phosphorylates GYS1 and GYS2 to inhibit their enzymatic activity in isolated muscle and liver treated with AMPK activators, thus reducing GYS-dependent glycogen synthesis [45, 46]. However, recent studies demonstrate that binding of G6P induced allosteric activation of GYS1 can overcome phosphorylation-mediated inhibition [36]. Indeed, chronic AMPK activation has been shown to promote glucose uptake and glycogen accumulation in skeletal and cardiac muscles [47], whereas knockout of AMPKβ2 subunit significantly reduces muscle glycogen content in mice [17]. Here, we show that Drp1-loss triggers activation of AMPK to promote glycogen synthesis by upregulating GYS1 mRNA expression. In addition, increased G6P production observed in Drp1 knockdown cells likely contributes to the activation of GYS enzyme activity directly. Together, mitochondrial dysregulation stimulates AMPK to favor the glycogenesis pathway in colon cancer cells. Interestingly, the binding of glycogen to the β-subunit of AMPK has been shown to inhibit its kinase activity [48]. It is likely that increased glycogen accumulation may progressively decrease AMPK activity during the tumorigenesis process. Consistent with this notion, we find that the phosphorylation levels of AMPK in xenograft tumor tissues derived from Drp1 knockdown cells are similar as that of control tumors (data not shown). Future studies are needed to assess the bi-direction regulation between AMPK and glycogen metabolism at different stages of tumorigenesis and progression.

Although downregulation of Drp1 decreases cell proliferation under regular growth conditions (Fig. 4) [10], Drp1 knockdown cells survive better when subjected to glucose deprivation conditions as compared to control cells. Results from our study support the hypothesis that increased glycogen serves as a glucose bank that can be utilized to support cancer cell survival when glucose is limited. Indeed, we show that glucose starvation depletes glycogen stores in both control and Drp1 knockdown cells, and higher levels of glycogen observed in Drp1 knockdown cells likely provide the metabolic substrate for a longer period. Furthermore, while control cells are largely resistant to DAB treatment, abrogation of glycogenolysis significantly decreases the survival of Drp1 knockdown cells. Similarly, it has been shown previously that knockdown of glycogen phosphorylase liver isoform (PYGL) sensitizes glioblastoma cells to radiation treatment [49]. It is of particular interest for future studies to determine if glycogen accumulation also provide survival advantage to cancer cells against other nutrient deprivation conditions.

In summary, our study uncovers a previously unrecognized mechanism by which Drp1-loss activates AMPK to stimulate glycogen accumulation. Using cancer cells, tumor organoids and xenograft tumor models, we show that downregulation Drp1 triggers a compensatory metabolic program to sustain cancer cell survival under stress conditions. Our data suggest that Drp1-targeted therapies are unlikely to be sufficient for eradicating cancer cells, however, inhibition of glycogenolysis may enhance chemosensitivity in colon cancer.

### Supplementary information

Supplemental materials and figures

Original Data File

Reproducibility checklist

## Data Availability

All data generated or analyzed during this study are available from the corresponding author on reasonable request.
