# Supplementary material for: Inhibition of mitochondrial fission activates glycogen synthesis to support cell survival in colon cancer
Source: Cell Death Dis. 2023 Oct 10;14(10):664. doi: 10.1038/s41419-023-06202-3 (PMC10564897; doi:10.1038/s41419-023-06202-3)
Supplement: Supplementary file 1 — Supplemental materials and figures [file 41419_2023_6202_MOESM1_ESM.docx]

**Inhibition of mitochondrial fission activates glycogen synthesis to support cell survival in colon cancer**

**Supplemental Materials**

**Table S1. Oligonucleotides utilized in RT-qPCR reactions**

|  | **Name** | **Forward (5’-3’)** | **Reverse (5’-3’)** |
| --- | --- | --- | --- |
| Human genes | *ACTB* | CATGTACGTTGCTATCCAGGC | CTCCTTAATGTCACGCACGAT |
|  | *GYS1* | GCGCTCACGTCTTCACTACTG | TCCAGATGCCCATAAAAATGGC |
| Mouse genes | *Actb* | GGCTGTATTCCCCTCCATCG | CCAGTTGGTAACAATGCCATGT |
|  | *Ccnd1* | TGACTGCCGAGAAGTTGTGC | CTCATCCGCCTCTGGCATT |
|  | *Gys1* | GAACGCAGTGCTTTTCGAGG | CCAGATAGTAGTTGTCACCCCAT |

**Supplemental Figures**

**Figure S1. Knockdown of Drp1 alters glucose metabolism in SW480 cells.** (A) Cell lysates of control (sh-Ctrl) and two Drp1 knockdown (sh-Drp1-B3 and sh-Drp1-B4) SW480 cells were analyzed for the expression of Drp1 and β-actin using western blot. (B) Control and Drp1 knockdown cells were subjected to Mito Stress tests using Seahorse XF96 Extracellular Flux Analyzer as described in Materials and Methods. The OCR measurements associated with basal, maximal, and spare capacity of mitochondrial respiration were calculated by normalizing to total cell numbers. Data represent the mean ± SD (n=10, ** p < 0.01). (C) Control and Drp1 knockdown cells were subjected to Glycolysis Stress tests using Seahorse XF96 Extracellular Flux Analyzer. The ECAR measurements associated with glycolysis, glycolytic capacity, and glycolytic reserve were calculated by normalizing to total cell numbers. Data represent the mean ± SD (n=10, ** p < 0.01 and **** p < 0.0001). (D) Control and Drp1 knockdown SW480 cells were incubated with 2-NBDG and Hoescht in low glucose media for 1 h. Relative glucose uptake was calculated by normalizing fluorescence signals detected from 2-NBDG to Hoescht. Data were presented as mean ± SD (n=4, * p < 0.05 and ** p < 0.01).

**Figure S2. Knockdown of Drp1 promotes glycogen accumulation in SW480 cells.** (A) Representative confocal images of control (sh-Ctrl) and Drp1 knockdown (sh-Drp1-B3 and sh-Drp1-B4) SW480 cells that were stained with antibodies against Drp1 (red) and glycogen (green). Scale Bar, 20 μm. (B) Cell lysates from sh-Ctrl and sh-Drp1 SW480 cells were analyzed for the expression of Drp1, GYS1 and β-actin using western blot. (C) Representative western blots as shown in (B) were quantified to determine the relative GYS1 levels by normalizing GYS1 to β-actin. Data were presented as mean ± SD (n=3, * p < 0.05). (D) sh-Ctrl and sh-Drp1 SW480 cells cultured in low glucose media were analyzed for the expression of GYS1 mRNA using RT-qPCR. Data were presented as mean ± SD (n=3, * p < 0.05).

**Figure S3. Knockdown of Drp1 increases GYS1 expression through AMPK-dependent transcriptional activation.** (A) Cell lysates of sh-Ctrl, sh-Drp1 SW480 cells were analyzed for the expression of Drp1, GYS1, phospho-AMPK (p-AMPK), total AMPK and β-actin using western blot. (B) Representative western blot as shown in (A) were quantified to determine the relative p-AMPK levels by normalizing p-AMPK to total AMPK. Data were presented as mean ± SD (n=3, ** p < 0.01). (C) Sh-Ctrl and sh-Drp1 SW480 cells were treated with DMSO or AMPK inhibitor compound C (AMPKi, 10 μM) for 24 h in low glucose media. Cell lysates were analyzed for the expression of Drp1, GYS1, p-AMPK, phospho-ACC (p-ACC), total ACC, total AMPK, and β-actin using western blot. (D) Representative western blots as shown in (C) were quantified to determine the relative GYS1 levels by normalizing GYS1 to β-actin. Data were presented as mean ± SD (n=3, * p < 0.05, ** p < 0.01, *** p < 0.001 and **** p < 0.0001). (E) The relative expression of GYS1 mRNA was determined using RT-qPCR in sh-Ctrl and sh-Drp1 SW480 cells treated with DMSO or AMPKi. Data were presented as mean ± SD (n=3, * p < 0.05, *** p < 0.001 and **** p < 0.0001). (F) Sh-Ctrl and sh-Drp1 SW480 cells were treated with DMSO or AMPKi in low glucose media for 24 h. Representative confocal images were obtained from cells stained with the anti-glycogen antibody. Scale Bar, 10 μm. (G) The relative fluorescence intensity of glycogen staining was quantified using ImageJ fluorescence analyzer. Data were presented as mean ± SD (n=20, * p <0.05 and **** p < 0.0001).

**Figure S4. Depletion of Drp1 in Apc-derived tumor organoids decreases cell proliferation without affecting colony formation.** (A) Apc and Apc/Drp1-KO organoids grown in 3D Matrigel for 3 days were analyzed for the expression of Ccnd1 mRNA using RT-qPCR. Data were presented as mean ± SD (n=6, *p < 0.05). (B) Total 10,000 single cells dissociated from Apc and Apc/Drp1-KO organoids were seeded in 3D Matrigel and cultured for 3 days. The number of colonies formed were counted and expressed as mean ± SD (n=6).

**Figure S5. Increased glycogen storage functions as a survival mechanism for Drp1 knockdown cells.** (A) Sh-Ctrl and sh-Drp1 SW480 cells were cultured in glucose-free medium for 72 h. The percentage of cell survival were obtained by normalizing the number of cells survived in glucose-free media to that of regular growth media. Data were presented as mean ± SD (n=7, **** p < 0.0001). (B) Sh-Ctrl and sh-Drp1 SW480 cells were cultured in low glucose or glucose-free media for 24 h. Representative confocal images were obtained from cells stained with the anti-glycogen antibody. Scale Bar, 10 μm. (C) The relative fluorescence intensity of glycogen staining was quantified using ImageJ fluorescence analyzer (n=20, * p < 0.05, ** p < 0.01 and **** p < 0.0001). (D) Sh-Ctrl and sh-Drp1 SW480 cells were treated with irinotecan, DAB or combinations of both agents for 72 h. Cells treated with DMSO were used as control. The relative of cell survival were obtained by normalizing to cells treated with DMSO. Data were presented as mean ± SD (n=6, * p < 0.05, ** p < 0.01, *** p < 0.001 and **** p < 0.0001).
