# Supplementary material for: Inhibition of mitochondrial fission activates glycogen synthesis to support cell survival in colon cancer
Source: Cell Death Dis. 2023 Oct 10;14(10):664. doi: 10.1038/s41419-023-06202-3 (PMC10564897; doi:10.1038/s41419-023-06202-3)

Figure 1A

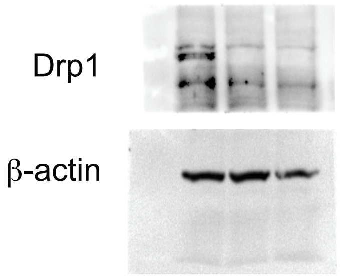

Figure 2B

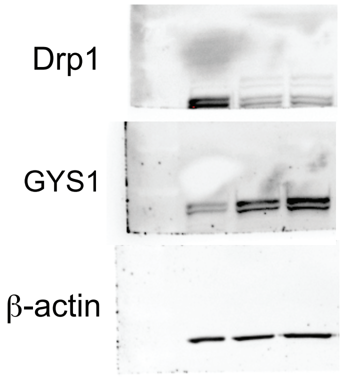

Figure 2E

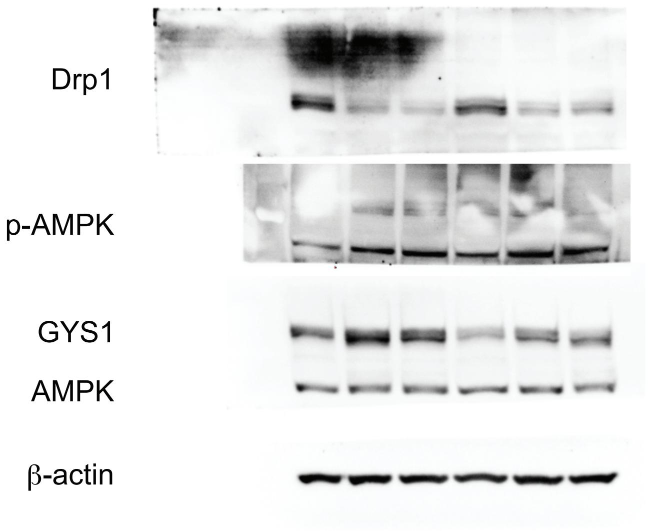

Figure 3A

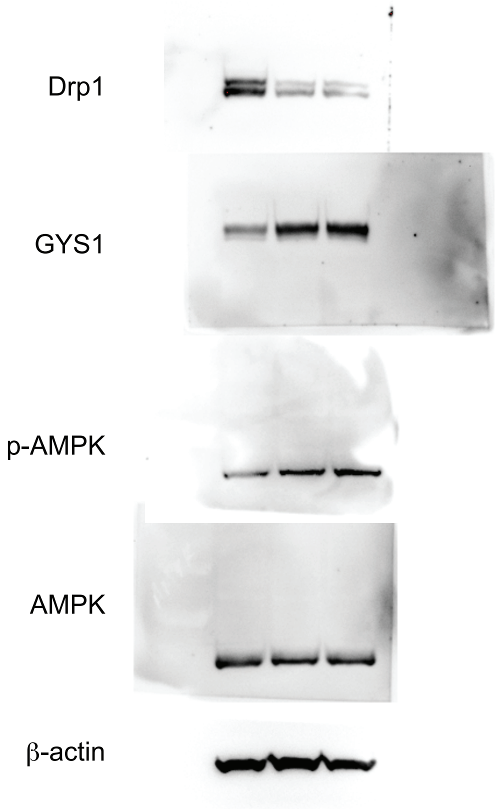

Figure 3C

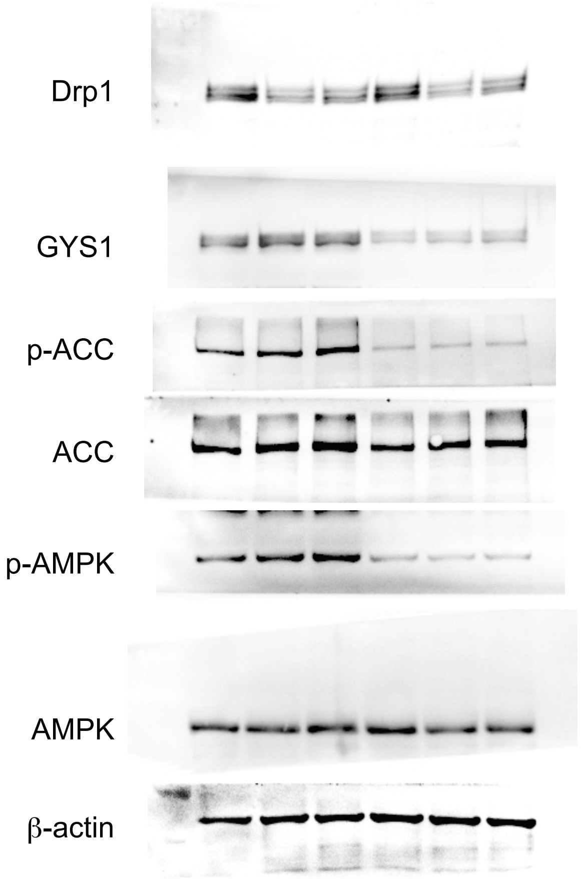

Figure 4A

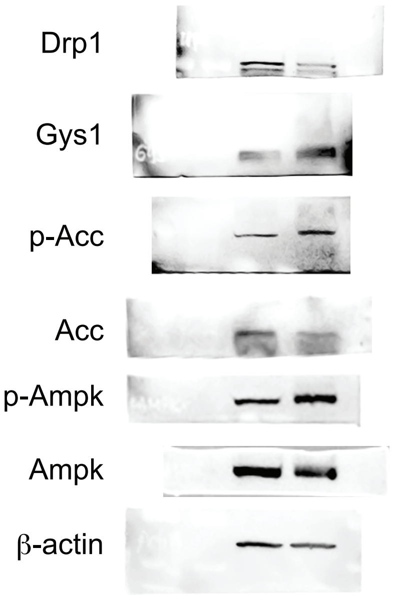

Figure 5A

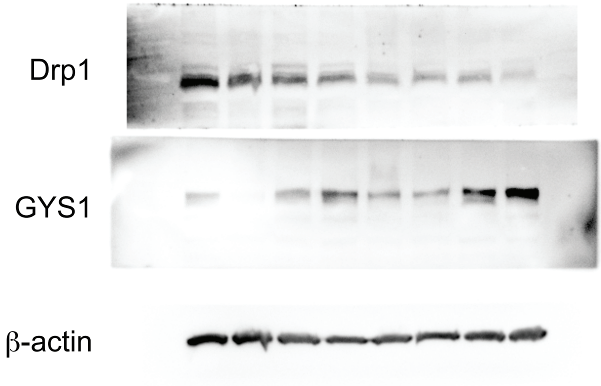

Figure S1A

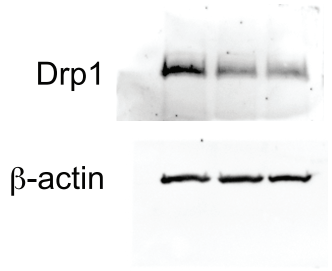

Figure S2B

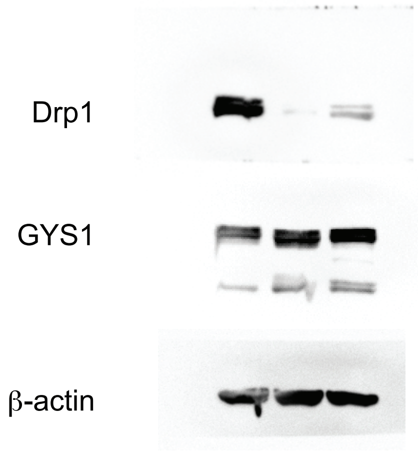

Figure S3A

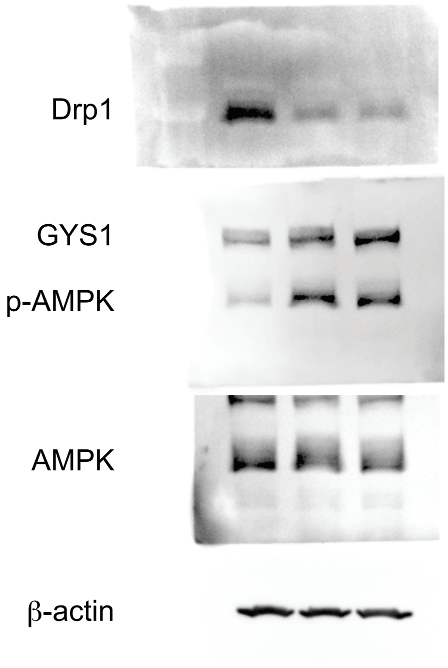

Figure S3C

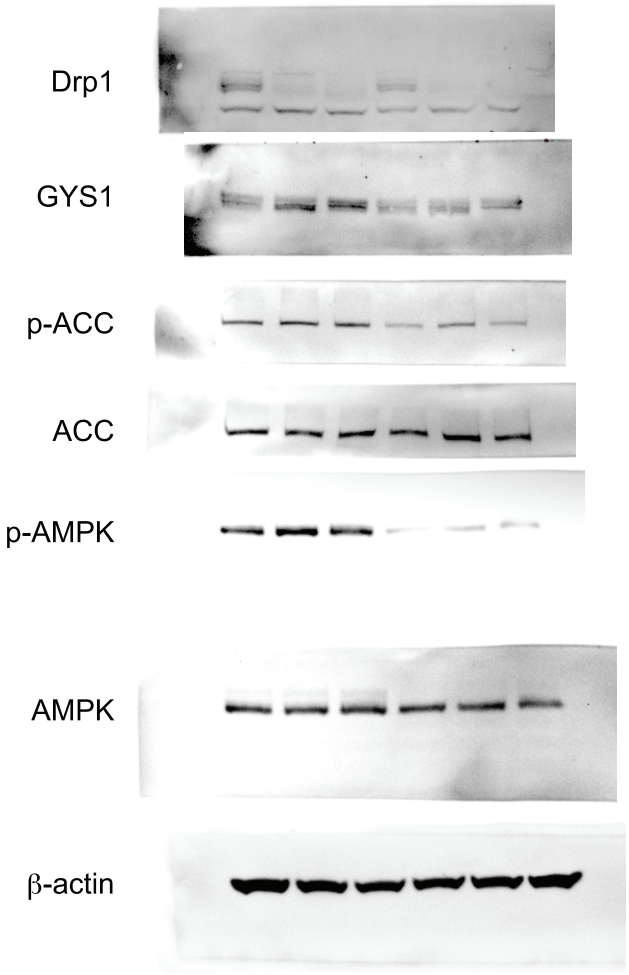

Supplement: Supplementary file 2 — Original Data File [file 41419_2023_6202_MOESM2_ESM.pdf]
